# Supplementary material for: Mitochondrial DNA Haplogroup N9a Negatively Correlates with Incidence of Hepatocellular Carcinoma in Northern China
Source: Mol Ther Nucleic Acids. 2019 Sep 12;18:332–40. doi: 10.1016/j.omtn.2019.09.001 (PMC6807372; doi:10.1016/j.omtn.2019.09.001)
Supplement: Document S1. Tables S1–S11 [file mmc1.pdf]

OMTN, Volume *18*

Supplemental Information

Mitochondrial DNA Haplogroup N9a Negatively Correlates with Incidence of Hepatocellular Carcinoma in Northern China

Shixuan Hua, Meinan Li, Qiongya Zhao, Junyi Wang, Yaping Zhou, Jiangtao Liu, Hezhi Fang, Minghua Jiang, and Lijun Shen

Table S1. Analysis of association between mitochondrial haplogroups and HBV/HCV positive HCC

MtDNA haplogroup	Patients (n = 338)	Controls (n = 511)	OR (95% CI)	*P* value
Macro-haplogroup				
A	29 (8.6)	42 (8.2)	0.890 (0.510-1.555)	0.683
B	52 (15.4)	72 (14.1)	0.986 (0.625-1.555)	0.951
D	111 (32.8)	140 (27.4)	1.0	
F	33 (9.8)	63 (12.3)	0.647 (0.389-1.075)	0.093
G	16 (4.7)	22 (4.3)	0.872 (0.425-1.788)	0.708
M7	22 (6.5)	35 (6.8)	0.756 (0.410-1.393)	0.370
M8	30 (8.9)	46 (9.0)	0.691 (0.402-1.187)	0.181
M9	3 (0.9)	6 (1.2)	0.600 (0.140-2.575)	0.492
M10	6 (1.8)	12 (2.3)	0.555 (0.196-1.572)	0.268
N9	6 (1.8)	35 (6.8)	0.191 (0.076-0.479)	0.000*
Others	30 (8.9)	38 (7.4)	0.962 (0.548-1.691)	0.894
Age			0.998 (0.987-1.010)	0.783
Gender			0.280 (0.202-0.388)	0.000*
Sub-haplogroup				
B4	32 (9.5)	36 (7.0)	1.167 (0.650-2.094)	0.605
B5	15 (4.4)	28 (5.5)	0.899 (0.434-1.863)	0.775
CZ	22 (6.5)	37 (7.2)	0.657 (0.352-1.228)	0.188
D4	77 (22.8)	101 (19.8)	1.0	
D5	32 (9.5)	38 (7.4)	1.151 (0.644-2.057)	0.636
F1	24 (7.1)	37 (7.2)	0.771 (0.417-1.425)	0.407
N9a	5 (1.5)	26 (5.1)	0.213 (0.077-0.590)	0.003*
Y	1 (0.3)	9 (1.8)	0.158 (0.019-1.317)	0.088
Others[a]	79 (23.4)	122 (23.9)	0.841 (0.548-1.291)	0.429
Age			0.999 (0.987-1.011)	0.869
Gender			0.281 (0.202-0.390)	0.000*

* indicated $P < 0.004$ (0.05/14), adjusted *P* value with Bonferroni correction while 14 haplogroups were studied; CI, confidence interval; OR, odds ratio; values in ( ) are the percentage (%) of samples; [a] Haplogroups with frequencies less than 5% in both patients and controls.

Table S2. Analysis of association between mitochondrial haplogroups and HBV/HCV negative HCC

MtDNA haplogroup	Patients (n = 43)	Controls (n = 511)	OR (95% CI)	*P* value
Macro-haplogroup				
A	2 (4.7)	42 (8.2)	0.385 (0.085-1.744)	0.215
B	2 (4.7)	72 (14.1)	0.240 (0.054-1.074)	0.062
D	17 (39.5)	140 (27.4)	1.0	
F	6 (14.0)	63 (12.3)	0.768 (0.287-2.059)	0.600
G	5 (11.6)	22 (4.3)	1.894 (0.626-5.730)	0.258
M7	2 (4.7)	35 (6.8)	0.460 (0.101-2.101)	0.316
M8	5 (11.6)	46 (9.0)	0.786 (0.272-2.274)	0.657
M9	0 (0.0)	6 (1.2)	0.000 (0.000)	0.999
M10	0 (0.0)	12 (2.3)	0.000 (0.000)	0.999
N9	2 (4.7)	35 (6.8)	0.403 (0.088-1.845)	0.242
Others	2 (4.7)	38 (7.4)	0.429 (0.094-1.948)	0.273
Age			1.018 (0.993-1.043)	0.160
Gender			0.470 (0.233-0.949)	0.035
Sub-haplogroup				
B4	2 (4.7)	36 (7.0)	0.404 (0.087-1.883)	0.248
B5	0 (0)	28 (5.5)	0.000 (0.000)	0.998
CZ	3 (7.0)	37 (7.2)	0.491 (0.132-1.834)	0.290
D4	14 (32.6)	101 (19.8)	1.0	
D5	3 (7.0)	38 (7.4)	0.497 (0.133-1.857)	0.298
F1	5 (11.6)	37 (7.2)	0.879 (0.292-2.644)	0.819
N9a	2 (4.7)	26 (5.1)	0.454 (0.096-2.156)	0.321
Y	0 (0.0)	9 (1.8)	0.000 (0.000)	0.999
Others[a]	5 (11.6)	100 (19.6)	0.334 (0.115-0.970)	0.044
Age			1.028 (0.995-1.046)	0.110
Gender			0.440 (0.237-0.970)	0.041

CI, confidence interval; OR, odds ratio; values in ( ) are the percentage (%) of samples; [a] Haplogroups with frequencies less than 5% in both patients and controls.

Table S3. False-positive report probability analysis for significant finding

SNP	OR (95%CI)	*P*	Statistical power[a]	Prior probability				
				**0.25**	**0.1**	0.01	0.001	0.0001
C16257A	0.290 (0.123-0.685)	0.005	0.802	**0.018**	**0.056**	0.308	0.856	0.978

CI, confidence interval; OR, odds ratio; [a] statistical power was computed by the OR and *p* value; the odds ratio of 0.2 for protective effect.

Table S4. Analysis of association between mitochondrial DNA SNPs and HBV/HCV positive HCC

mtSNP	Patients (n = 338)	Controls (n = 511)	OR (95% CI)	*P* value
m.150C>T	64 (18.9)	94 (18.4)	1.076 (0.780-1.484)	0.656
m.249delA	42 (12.4)	85 (16.6)	0.647 (0.446-0.941)	0.023
m.10397A>G	34 (10.1)	38 (7.4)	1.559 (1.007-2.416)	0.047
m.10398A>G	224 (66.3)	316 (61.8)	1.220 (0.936-1.590)	0.140
m.10400C>T	202 (59.8)	278 (54.4)	1.147 (0.888-1.480)	0.294
m.16129G>A	54 (16.0)	106 (20.7)	0.649 (0.462-0.912)	0.013
m.16189T>C	124 (36.7)	147 (28.8)	1.486 (1.137-1.943)	0.004*
m.16223C>T	211 (62.4)	293 (57.3)	1.174 (0.906-1.521)	0.224
m.16257C>A	5 (1.5)	26 (5.1)	0.262 (0.107-0.643)	0.003*
m.16362T>C	165 (48.8)	214 (41.9)	1.317 (1.021-1.698)	0.034

*indicated $P < 0.005$ (0.05/10), adjusted *P* value with Bonferroni correction while 10 SNPs were studied; CI, confidence interval; OR, odds ratio; values in ( ) are the percentage (%) of samples.

Table S5. Analysis of association between mitochondrial DNA SNPs and HBV/HCV negative HCC

mtSNP	Patients (n = 43)	Controls (n = 511)	OR (95% CI)	*P* value
m.150C>T	3 (0.07)	94 (18.4)	0.262 (0.080-0.857)	0.027
m.249delA	6 (14.0)	85 (16.6)	0.858 (0.392-1.875)	0.700
m.10397A>G	3 (7.0)	38 (7.4)	0.951 (0.326-2.776)	0.927
m.10398A>G	26 (60.5)	316 (61.8)	1.049 (0.589-1.868)	0.870
m.10400C>T	26 (60.5)	278 (54.4)	1.449 (0.815-2.577)	0.206
m.16129G>A	7 (16.3)	106 (20.7)	0.754 (0.360-1.582)	0.456
m.16189T>C	10 (23.3)	147 (28.8)	0.728 (0.372-1.424)	0.354
m.16223C>T	27 (62.8)	293 (57.3)	1.413 (0.787-2.539)	0.247
m.16257C>A	2 (4.7)	26 (5.1)	0.674 (0.155-2.928)	0.598
m.16362T>C	22 (51.2)	214 (41.9)	1.665 (0.951-2.915)	0.074

CI, confidence interval; OR, odds ratio; values in ( ) are the percentage (%) of samples.

Table S6. Association between mtDNA haplogroups and serum AST level

MtDNA haplogroup	AST rise		Multivariate	
	Yes (n = 185) [n (%)]	No (n = 195) [n (%)]	OR (95% CI)	*P* value
A	15 (8.1)	16 (8.2)	1.097 (0.480-2.508)	0.827
B4	17 (9.2)	17 (8.7)	1.202 (0.541-2.671)	0.651
B5	7 (3.8)	8 (4.1)	1.031 (0.339-3.134)	0.957
CZ	15 (8.1)	10 (5.1)	1.779 (0.715-4.428)	0.215
D4	40 (21.6)	51 (26.2)	1.0	
D5	19 (10.3)	16 (8.2)	1.413 (0.637-3.132)	0.395
F1	16 (8.6)	13 (6.7)	1.602 (0.684-3.750)	0.278
G	7 (3.8)	14 (7.2)	0.701 (0.256-1.916)	0.488
M7	12 (6.5)	12 (6.2)	1.179 (0.472-2.943)	0.724
N9a	3 (1.6)	4 (2.1)	1.058 (0.222-5.039)	0.944
Y	0 (0.0)	1 (0.5)	0.000 (0.000)	1.000
Others	34 (18.4)	33 (16.9)	1.309 (0.689-2.487)	0.411
Age			0.978 (0.959-0.997)	0.023
HBV/HCV			1.354 (0.690-2.655)	0.378
Gender			1.072 (0.633-1.815)	0.795

CI, confidence interval; OR, odds ratio; eight patients are not available for AST clinical data.

Table S7. Association between mtDNA haplogroups and serum ALT level.

MtDNA haplogroup	ALT rise		Multivariate	
	Yes (n = 120) [n (%)]	No (n = 259) [n (%)]	OR (95% CI)	*P* value
A	11 (9.2)	19 (7.3)	1.228 (0.511-2.950)	0.646
B4	12 (10.0)	22 (8.5)	1.157 (0.500-2.675)	0.734
B5	5 (4.2)	10 (3.9)	1.087 (0.334-3.533)	0.890
CZ	9 (7.5)	16 (6.2)	1.106 (0.434-2.817)	0.833
D4	29 (24.2)	62 (23.9)	1.0	
D5	9 (7.5)	26 (10.0)	0.751 (0.309-1.826)	0.528
F1	8 (6.7)	21 (8.1)	0.761 (0.300-1.931)	0.565
G	5 (4.2)	16 (6.2)	0.692 (0.229-2.089)	0.513
M7	7 (5.8)	17 (6.6)	0.892 (0.330-2.408)	0.821
N9a	2 (1.7)	5 (1.9)	0.854 (0.154-4.734)	0.857
Y	0 (0.0)	1 (0.4)	0.000 (0.000)	1.000
Others	23 (19.2)	44 (17.0)	1.147 (0.582-2.257)	0.692
Age			0.990 (0.970-1.010)	0.322
HBV/HCV			1.022 (0.490-2.127)	0.955
Gender			0.577 (0.315-1.057)	0.075

CI, confidence interval; OR, odds ratio; nine patients are not available for ALT clinical data.

Table S8. Association between mtDNA haplogroups and serum ALB level.

MtDNA haplogroup	ALB down		Multivariate	
	Yes (n = 178) [n (%)]	No (n = 202) [n (%)]	OR (95% CI)	*P* value
A	13 (7.3)	18 (8.9)	0.975 (0.424-2.241)	0.953
B4	18 (10.1)	16 (7.9)	1.500 (0.676-3.330)	0.319
B5	7 (3.9)	8 (4.0)	1.180 (0.390-3.571)	0.769
CZ	13 (7.3)	12 (5.9)	1.439 (0.588-3.524)	0.425
D4	40 (22.5)	51 (25.2)	1.0	
D5	20 (11.2)	15 (7.4)	1.809 (0.816-4.013)	0.145
F1	9 (5.1)	20 (9.9)	0.567 (0.231-1.389)	0.214
G	7 (3.9)	14 (6.9)	0.588 (0.215-1.607)	0.301
M7	15 (8.4)	9 (4.5)	2.226 (0.876-5.657)	0.093
N9a	7 (3.9)	0 (0.0)	1912182463 (0.000)	0.999
Y	1 (0.6)	0 (0.0)	2526260229 (0.000)	1.000
Others	28 (15.7)	39 (19.3)	0.914 (0.480-1.741)	0.785
Age			1.018 (0.998-1.038)	0.076
HBV/HCV			0.822 (0.415-1.628)	0.575
Gender			0.902 (0.530-1.536)	0.704

CI, confidence interval; OR, odds ratio; eight patients are not available for ALB clinical data.

Table S9. Association between mtDNA haplogroups and serum TBIL level.

MtDNA haplogroup	TBIL rise		Multivariate	
	Yes (n = 86) [n (%)]	No (n = 294) [n (%)]	OR (95% CI)	*P* value
A	7 (8.1)	24 (8.2)	0.902 (0.336-2.421)	0.838
B4	9 (10.5)	25 (8.5)	1.133 (0.454-2.825)	0.789
B5	4 (4.7)	11 (3.7)	1.109 (0.313-3.925)	0.873
CZ	5 (5.8)	20 (6.8)	0.738 (0.243-2.236)	0.591
D4	21 (24.4)	70 (23.8)	1.0	
D5	3 (3.5)	32 (10.9)	0.294 (0.081-1.070)	0.063
F1	7 (8.1)	22 (7.5)	1.018 (0.377-2.751)	0.972
G	4 (4.7)	17 (5.8)	0.886 (0.263-2.985)	0.845
M7	5 (5.8)	19 (6.5)	0.839 (0.275-2.555)	0.757
N9a	3 (3.5)	4 (1.4)	2.984 (0.594-14.978)	0.184
Y	1 (1.2)	0 (0.0)	7065260239 (0.000)	1.000
Others	17 (19.8)	50 (17.0)	1.120 (0.531-2.363)	0.766
Age			0.985 (0.962-1.008)	0.200
HBV/HCV			2.400 (0.882-6.530)	0.087
Gender			0.590 (0.289-1.203)	0.147

CI, confidence interval; OR, odds ratio; eight patients are not available for TBIL clinical data.

Table S10. Characteristics of the participants

Characteristic	Patients (n = 388)	Controls (n = 511)
Age (years)		
10-20	1 (0.3)	1 (0.2)
21-30	3 (0.8)	21 (4.1)
31-40	24 (6.2)	55 (10.8)
41-50	103 (26.5)	77 (15.1)
51-60	123 (31.7)	136 (26.6)
61-70	103 (26.5)	152 (29.7)
71-80	27 (7.0)	58 (11.4)
81-90	4 (1.0)	11 (2.2)
Gender		
Male	308 (79.4)	282 (55.2)
Female	80 (20.6)	229 (44.8)

values in ( ) are the percentage (%) of samples

Table S11. Statistics of Han populations from different regions

Geography	Population ID	Locality	No. of samples	References
Northern China	HCC	Zhengzhou, Henan	388	This study
	CON	Zhengzhou, Henan	511	This study
	HA	Henan	198	[1]
	YB	Yanbian, Jilin	51	[2]
	GS	Gansu	44	[2]
	DL	Dalian, Liaoning	50	[2]
	FC	Fengcheng, Liaoning	50	[2]
	CF	Chifeng, Neimeng	44	[2]
	XN	Xining, Qinghai	41	[2]
	QD	Qingdao, Shandong	50	[2]
	TA	Taian, Shandong	76	[2]
	XA	Xi'an, Shanxi	53	[2]
	UR	Urumqi, Xinjiang	47	[2]
	QH	Qinghai	78	[3]
	ZB	Zibo, Shandong	50	[3]
Southern China	HF	Hefei, Anhui	42	[2]
	CT	Changtin, Fujian	54	[2]
	GZ	Guangzhou, Guangdong	68	[2]
	ZJ	Zhanjiang, Guangdong	30	[2]
	TL	Tianlin, Guangxi	26	[2]
	WH	Wuhan, Hubei	42	[2]
	CS	Changsha, Hunan	16	[2]
	NJ	Nanjing, Jiangsu	67	[2]
	NC	Nanchang, Jiangxi	23	[2]
	WC	Weicheng, Sichuan	70	[2]
	KM	Kunming, Yunnan	42	[2]
	YNHZ	Huize, Yunnan	59	[2]
	HK	HongKong	20	[3]
	TW1	Taiwan	66	[3]
	TW2	Taiwan	155	[3]
	SH	Shanghai	120	[3]
	ZJWZ	Wenzhou, Zhejiang	974	[4]

1. Su B, Xiao C, Deka R, Seielstad MT, Kangwanpong D, Xiao J, Lu D, Underhill P, Cavalli-Sforza L, Chakraborty R *et al*: *Y chromosome haplotypes reveal prehistorical migrations to the Himalayas. Human genetics* 2000, **107**(6):582-590.
2. Wen B, Li H, Lu D, Song X, Zhang F, He Y, Li F, Gao Y, Mao X, Zhang L *et al*: *Genetic evidence supports demic diffusion of Han culture. Nature* 2004, **431**(7006):302-305.
3. Yao YG, Kong QP, Bandelt HJ, Kivisild T, Zhang YP: *Phylogeographic differentiation of mitochondrial DNA in Han Chinese. Am J Hum Genet* 2002, **70**(3):635-651.
4. Fang H, Hu N, Zhao Q, Wang B, Zhou H, Fu Q, Shen L, Chen X, Shen F, Lyu J: *mtDNA Haplogroup N9a Increases the Risk of Type 2 Diabetes by Altering Mitochondrial Function and Intracellular Mitochondrial Signals. Diabetes* 2018, **67**(7):1441-1453.
