# Supplementary material for: Mitochondrial DNA Haplogroup N9a Negatively Correlates with Incidence of Hepatocellular Carcinoma in Northern China
Source: Mol Ther Nucleic Acids. 2019 Sep 12;18:332–40. doi: 10.1016/j.omtn.2019.09.001 (PMC6807372; doi:10.1016/j.omtn.2019.09.001)
Supplement: Document S2. Article plus Supplemental Information [file mmc5.pdf]

Molecular Therapy
Nucleic Acids
Original Article

AMERICAN SOCIETY of GENE & CELL THERAPY

Mitochondrial DNA Haplogroup N9a Negatively Correlates with Incidence of Hepatocellular Carcinoma in Northern China

Shixuan Hua,[1,2,7] Meinan Li,[3,7] Qiongya Zhao,[3,4,7] Junyi Wang,[3] Yaping Zhou,[3] Jiangtao Liu,[5] Hezhi Fang,[3] Minghua Jiang,[6] and Lijun Shen[3]

[1]Department of Laboratory Medicine, Henan Provincial People's Hospital, Zhengzhou, Henan, China; [2]Department of Laboratory Medicine, Fuwai Central China Cardiovascular Hospital, Zhengzhou, Henan, China; [3]Key Laboratory of Laboratory Medicine, Ministry of Education, Zhejiang Provincial Key Laboratory of Medical Genetics, College of Laboratory Medicine and Life Sciences, Wenzhou Medical University, Wenzhou, Zhejiang, China; [4]College of Laboratory Medicine, Hangzhou Medical College, Hangzhou, Zhejiang, China; [5]Department of Orthopedics Surgery, Ningbo No.2 Hospital, Ningbo, Zhejiang, China; [6]Department of Laboratory Medicine, the Second Affiliated Hospital, Wenzhou Medical University, Wenzhou, Zhejiang, China

Mitochondrial DNA (mtDNA) haplogroups are associated with various types of cancer; however, the molecular mechanisms by which mtDNA haplogroups affect primary hepatocellular carcinoma (HCC) are not known. In this study, we carried out a case-control study on 388 HCC patients and 511 geographically matched asymptomatic control subjects in northern China. We found that mtDNA haplogroup N9a and its diagnostic SNP, m.16257C > A, negatively correlated with the incidence of HCC in northern China (odds ratio [OR] 0.290, 95% confidence interval [CI] 0.123–0.685, p = 0.005), particularly in patients with infection of hepatitis B/C virus (HBV/HCV) (for haplogroup N9a: OR 0.213, 95% CI 0.077–0.590, p = 0.003; for m.16257C > A: OR 0.262, 95% CI 0.107–0.643, p = 0.003). However, mtDNA haplogroup N9a is not associated with clinical characteristics of HCC including serum alpha-fetoprotein (AFP) level and tumor size. In addition, cytoplasmic hybrid (cybrid) cells with N9a haplogroup (N9a10a and N9a1) had transcriptome profiles distinct from those with non-N9a (B5, D4, and D5) haplogroups. Gene set enrichment analysis (GSEA) showed that metabolic activity varied significantly between N9a and non-N9a haplogroups. Moreover, cells with haplogroup N9a negatively correlated with cell division and multiple liver cancer pathways compared with non-N9a cells. Although it is still unclear how N9a affects the aforementioned GSEA pathways, our data suggest that mtDNA haplogroup N9a is negatively correlated with the incidence and progression of HCC in northern China.

INTRODUCTION

In China, liver cancer is the second most common malignancy, with an estimated 360,000 new cases and 350,000 deaths per year.[1] Hepatocellular carcinoma (HCC) is the most common subtype of primary liver cancer (>80%).[2] Most HCC is caused by chronic liver injury due to hepatitis B/C virus (HBV/HCV) infection and alcohol abuse. Although the molecular mechanisms underlying the development and progression of HCC are poorly defined, functional studies suggest that both HBV/HCV infection and alcohol abuse are closely associated with mitochondrial dysregulation,[3,4] which has long been associated with the development of HCC.[5,6]

Mitochondria generate the energy necessary to sustain all cellular activities. Human mitochondria contain approximately 1,500 proteins, of which 13 are encoded by mtDNA. mtDNA variations are associated with multiple human conditions such as aging,[7] cancer,[8] and rare mitochondrial diseases.[9] Moreover, mtDNA haplogroups, specific mtDNA genetic variations defined by germline mtDNA mutations retained through evolution, are associated with cancer[10] and type 2 diabetes,[11] as well as neurodegenerative diseases, such as Leber hereditary optic neuropathy[12,13] and Alzheimer's disease.[14] Mechanistically, different mtDNA haplogroups can differentially affect mitochondrial performance. For example, haplogroups M and N have different mitochondrial matrix pH and intracellular calcium levels;[15] haplogroup J is more transcriptionally efficient and displays a higher level of mtDNA replication than haplogroup H.[16] Transcriptome analysis revealed significant differences in mRNA levels among cytoplasmic hybrid (cybrid) cells of different mtDNA haplogroups,[11] and by using mouse models of various mtDNA haplogroups, A.

Received 18 March 2019; accepted 5 September 2019;
https://doi.org/10.1016/j.omtn.2019.09.001.

[7]These authors contributed equally to this work.

Correspondence: Hezhi Fang, Key Laboratory of Laboratory Medicine, Ministry of Education, Zhejiang Provincial Key Laboratory of Medical Genetics, College of Laboratory Medicine and Life Sciences, Wenzhou Medical University, Wenzhou, Zhejiang, China.
E-mail: fangh@wmu.edu.cn
Correspondence: Minghua Jiang, Department of Laboratory Medicine, the Second Affiliated Hospital, Wenzhou Medical University, Wenzhou, Zhejiang, China.
E-mail: minghua93@126.com
Correspondence: Lijun Shen, Key Laboratory of Laboratory Medicine, Ministry of Education, Zhejiang Provincial Key Laboratory of Medical Genetics, College of Laboratory Medicine and Life Sciences, Wenzhou Medical University, Wenzhou, Zhejiang, China.
E-mail: ysslj23@163.com

 © 2019 The Author(s).
This is an open access article under the CC BY license (http://creativecommons.org/licenses/by/4.0/).

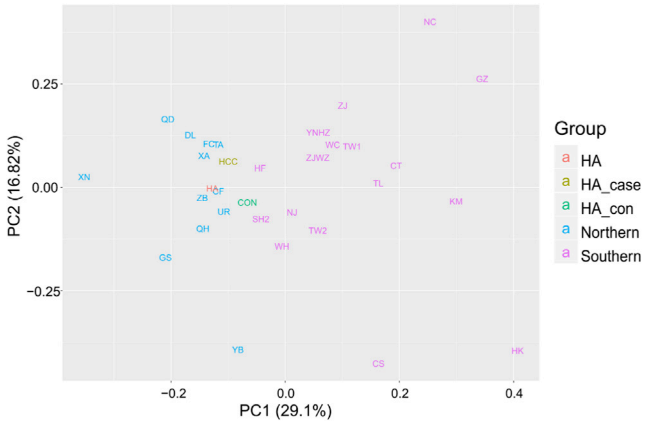

Figure 1. The PCA of HCC Patients and Controls, as well as Other Reported Han Populations across China, Based on mtDNA Haplogroup Frequencies
HCC patients and controls are from Zhengzhou, Henan, are shown with yellow and green fonts, the previously reported populations from Henan are shown with red fonts, and the previously reported populations from northern and southern China are shown with blue and purple fonts.

Latorre-Pellicer et al. saw that liver function varied significantly among mice with different mtDNA genetic backgrounds.[17]

Three recent studies, including participants from northern China (Hebei),[18] eastern China (Shanghai),[19] and southern China (Guangxi),[20] demonstrated that distinct mtDNA haplogroups are associated with various HCC-associated factors.[18–20] Notably, effects of mtDNA haplogroup defining SNPs on the prognosis of HCC were conflicted,[19,20] and the correlations between specific mtDNA haplogroup and the incidence of HCC were not ever investigated in these studies. Multiple factors may contribute to this unexpected finding. Different environmental conditions among participants may have differentially affected the role of mtDNA haplogroups in HCC.

Pitfalls including insufficient or inappropriate recruitment criteria of participants, improper choice of statistical analysis methods, and lack of replication cohort may have also cast doubt on the validity of the conclusion.[21,22] In addition, two of the studies included patients with small sample size in their analyses;[18,20] therefore, their findings may not reflect the potential association of mtDNA SNPs/haplogroups with HCC-associated factors. To date, only the study using eastern Chinese population adopted a considerable number of patients to analyze the relationships between mtDNA haplogroup and HCC.[19] To verify and to further assess the possible contribution of mtDNA haplogroup on the prevalence of HCC, we conducted a cohort study with participants from northern China to reevaluate the potential correlation between mtDNA haplogroups and the incidence and severity of HCC.

RESULTS

mtDNA Haplogroup N9a Is Associated with Decreased Risk of HCC

To test whether there is a population stratification between the patients and control subjects, which may tone down the confidence level of the study, we performed a principal-component analysis (PCA) for patients and control subjects based on mtDNA haplogroup frequencies as previously described.[23] As shown in Figure 1, both patients and control subjects are close with each other, and were co-clustered with most of the northern Chinese population. This result suggested that there is no population stratification between the patients and control subjects.

Table 1. Analysis of Association between Macro-Haplogroup M/N and HCC

Macro-Haplogroup	Patients (n = 388)	Controls (n = 511)	OR (95% CI)	p Value
M	229 (59.0)	278 (54.4)	1.154 (0.875–1.520)	0.310
N	159 (41.0)	233 (45.6)		
Age			1.000 (0.989–1.011)	0.992
Gender			0.322 (0.238–0.436)	0.000*

*p < 0.025 (0.05/2), adjusted p value with Bonferroni correction while two haplogroups were studied. Values in parentheses are the percentage of samples. CI, confidence interval; OR, odds ratio.

While mtDNA haplogroup M was found to be associated with late onset of HCC in northern China with limited sample size,[18] we first asked whether the frequency of haplogroup M associated with HCC in larger sample size. However, we found that haplogroup M is not associated with HCC with adjustment of age and sex (Table 1) and is not associated with HCC at late onset by using the Kaplan-Meier method (Figure 2). Furthermore, no significant difference was found in the prevalence of macro-haplogroups A, B, D, F, G, and M7–10 between patients and controls (Table 2). We did, however, find that the prevalence of haplogroup N9 was significantly lower in patients with HCC compared to controls, using multivariate logistic regression analysis with adjustment for age and sex (odds ratio [OR] 0.241, [95% confidence interval (CI) 0.110–0.531], p < 0.001) (Table 2). Further analysis of sub-haplogroups from ten macro-haplogroups revealed that sub-haplogroup N9a (OR 0.248, [95% CI 0.101–0.610], p = 0.002) but not sub-haplogroup Y of N9 contributes to the negative association between macro-haplogroup N9 and HCC (Table 2).

To ask whether HBV/HCV infection plays a role in haplogroup-associated HCC occurrence, we divided patients into HBV/HCV-positive and HBV/HCV-negative groups and compared them with the control group separately. As shown in Table S1, haplogroup N9 (OR 0.191, [95% CI 0.076–0.479], p < 0.001) and its sub-haplogroup N9a (OR 0.213, [95% CI 0.077–0.590], p = 0.003) are negatively associated with the occurrence of HBV/HCV-positive HCC. However, haplogroup N9 and its sub-haplogroup N9a are not associated with the occurrence of HBV/HCV-negative HCC (Table S2).

Taken together, we conclude that there is a significant negative correlation between haplogroup N9a and the prevalence of HBV/HCV-positive HCC in the study participants. Because only a small portion of patients (n = 43) with HCC were diagnosed without HBV/HCV infection, further studies with a greater number of HBV/HCV-negative HCC patients are needed to fully understand the role of mtDNA haplogroups in HBV/HCV-free HCC.

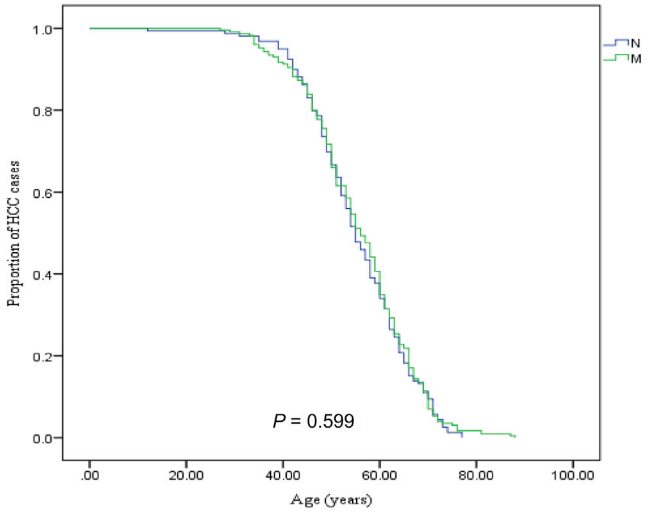

Figure 2. The Association between Macro-Haplogroup M/N and Age at Onset HCC

p value was estimated by Kaplan-Meier method.

Table 2. Analysis of Association between Mitochondrial Haplogroups and HCC

mtDNA Haplogroup	Patients (n = 388)	Controls (n = 511)	OR (95% CI)	p Value
Macro-haplogroup				
A	31 (8.0)	42 (8.2)	0.800 (0.465–1.375)	0.419
B	57 (14.7)	72 (14.1)	0.906 (0.584–1.406)	0.660
D	129 (33.2)	140 (27.4)	1.0	
F	41 (10.6)	63 (12.3)	0.680 (0.422–1.095)	0.112
G	21 (5.4)	22 (4.3)	0.997 (0.511–1.947)	0.994
M7	24 (6.2)	35 (6.8)	0.713 (0.394–1.289)	0.263
M8	35 (9.0)	46 (9.0)	0.695 (0.414–1.165)	0.167
M9	3 (0.8)	6 (1.2)	0.503 (0.118–2.143)	0.352
M10	6 (1.5)	12 (2.3)	0.475 (0.169–1.339)	0.159
N9	9 (2.3)	35 (6.8)	0.241 (0.110–0.531)	0.000*
Others	32 (8.2)	38 (7.4)	0.889 (0.514–1.540)	0.676
Age			1.001 (0.990–1.012)	0.876
Gender			0.304 (0.223–0.413)	0.000*
Sub-haplogroup				
B4	36 (9.3)	36 (7.0)	1.099 (0.626–1.928)	0.743
B5	16 (4.1)	28 (5.5)	0.776 (0.384–1.571)	0.482
CZ	25 (6.4)	37 (7.2)	0.637 (0.350–1.159)	0.140
D4	91 (23.5)	101 (19.8)	1.0	
D5	36 (9.3)	38 (7.4)	1.091 (0.624–1.907)	0.759
F1	30 (7.7)	37 (7.2)	0.811 (0.455–1.445)	0.477
N9a	7 (1.8)	26 (5.1)	0.248 (0.101–0.610)	0.002*
Y	2 (0.5)	9 (1.8)	0.253 (0.051–1.246)	0.091
Others[a]	69 (17.8)	100 (19.6)	0.753 (0.488–1.162)	0.200
Age			1.001 (0.990–1.013)	0.795
Gender			0.307 (0.225–0.417)	0.000*

*p < 0.004 (0.05/14), adjusted p value with Bonferroni correction while 14 haplogroups were studied; values in parentheses are the percentage of samples. CI, confidence interval; OR, odds ratio.

[a]Haplogroups with frequencies less than 5% in both patients and controls.

mtDNA SNP Analysis of Patients with HCC

To determine the prevalence of distinct mitochondrial SNPs (mtSNPs) in patient and control groups, we used ten SNPs that are preferentially expressed by distinct haplogroups (i.e., defining SNPs). To test whether the sample size we studied here was large enough, we determined a minimal sample size that provides sufficient statistical power to detect the OR = 0.5 or OR = 2 with the following conditions: average population minimum allele frequency (MAF) = 20% (average MAF in control group from this study is 31.24%) and case/control ratio = 1, p = 0.05, power = 90%. The calculated minimal sample size was 217 for OR < 0.5 and 180 for OR > 2, indicating that the sample size we used in this analysis (388) was qualified. After adjusting for age and sex, we found that the frequency of m.16257C > A, a defining mtSNP of the N9a haplogroup, was significantly lower in patients than in control participants (OR 0.290, [95% CI 0.123–0.685], p = 0.005) (Table 3). To exclude the false-positive probability of this significant association between m.16257C > A and HCC, a false-positive report probability (FPRP) analysis was used. As shown in Table S3, our results showed that with prior probability of 0.25 and 0.1, FPRP values were less than their significant predetermined value (0.2). This result suggested that our findings are deserving of attention. In addition, m.16189T > C, one of the common mtSNPs, was found higher in patients than in control participants (OR 1.497, [95% CI 1.115–2.010], p = 0.007); however, after applying the Bonferroni correction to adjust for ten simultaneous SNP statistical analysis, only p < 0.005 (0.05/10) was considered statistically significant (see Table 3). By dividing the patients into HBV/HCV-positive and HBV/HCV-negative groups, we found that both m.16189T > C (OR 1.486, [95% CI 1.137–1.943], p = 0.004) (Table S4) and m.16257C > A (OR 0.262, [95% CI 0.107–0.643], p = 0.003) (Table S4) are positively and negatively associated with the occurrence of HBV/HCV-positive HCC, respectively. However, m.16189T > C and m.16257C > A were found to not be associated with the occurrence of HBV/HCV-negative HCC (Table S5). The prevalence of other mtSNPs we evaluated, including the macro-haplogroup M-defining mtSNP, m.10400C > T, and the haplogroup D5a-defining mtSNP, m.10397A > G, were similar in patient and control groups regardless of the HBV/HCV infection status. However, further studies with a greater number of HBV/HCV-negative HCC patients are needed to fully understand the role of mtDNA in HBV/HCV-free HCC.

These data indicate that m.16189T > C and m.16257C > A are positively and negatively associated with the prevalence of HBV/HCV-positive HCC, respectively. The associations between mtSNPs and HBV/HCV-negative HCC are currently not known, and further

Table 3. Analysis of Association between Mitochondrial DNA SNPs and HCC

mtSNP	Patients (n = 388)	Controls (n = 511)	OR (95% CI)	p Value
m.150C > T	68 (17.5)	94 (18.4)	0.958 (0.671–1.367)	0.813
m.249delA	50 (12.9)	85 (16.6)	0.703 (0.477–1.037)	0.076
m.10397A > G	38 (9.8)	38 (7.4)	1.426 (0.875–2.324)	0.155
m.10398A > G	254 (65.5)	316 (61.8)	1.189 (0.895–1.580)	0.232
m.10400C > T	229 (59.0)	278 (54.4)	1.154 (0.875–1.520)	0.310
m.16129G > A	63 (16.2)	106 (20.7)	0.702 (0.492–1.001)	0.051
m.16189T > C	138 (35.6)	147 (28.8)	1.497 (1.115–2.010)	0.007
m.16223C > T	238 (61.3)	293 (57.3)	1.066 (0.806–1.410)	0.652
m.16257C > A	7 (1.8)	26 (5.1)	0.290 (0.123–0.685)	0.005*
m.16362T > C	188 (48.5)	214 (41.9)	1.293 (0.983–1.702)	0.066

*p < 0.005 (0.05/10), adjusted p value with Bonferroni correction while 10 SNPs were studied. CI, confidence interval; OR, odds ratio. Values in parentheses are the percentage of samples.

studies with greater number of HBV/HCV-negative HCC patients are needed.

Evaluation of Clinical Characteristics Associated with Distinct mtDNA Haplogroups

To evaluate whether distinct mtDNA haplogroups were associated with specific clinical characteristics of HCC, we analyzed several clinical characteristics including alpha-fetoprotein (AFP) levels, tumor size, and biomarkers of liver function such as aspartate aminotransferase (AST), alanine aminotransferase (ALT), serum albumin (ALB), and total bilirubin (TBIL) levels. We did not correct for differences in HBV infection status and tumor metastasis among haplogroups, because only a small percentage of patients tested negative for HBV infection (15.49%) and tumor metastasis (7.94%). With our original number of recruited participants, dividing haplogroups further based on these two characteristics would diminish the power of our study. A recent study found that liver function biomarkers were elevated in patients with HCC,[24] suggesting that biomarkers of liver function may be closely associated with the risk of developing HCC. However, using multivariate logistical regression analysis with adjustment for age, sex, and HBV/HCV infection, we found no correlation between mtDNA haplogroups and AST (Table S6), ALT (Table S7), ALB (Table S8), or TBIL (Table S9). Next, we analyzed the potential association between mtDNA haplogroups and AFP expression in patients with HCC. Based on the criterion that AFP > 400 ng/mL is a diagnostic index of HCC,[25] patients were divided into high AFP expression group (>400 ng/mL) and low AFP expression group (<400 ng/mL); however, haplogroups including N9a were not associated with the level of AFP expression level (Table 4). By dividing the patients into group with large tumor size (diameter > 5 cm) and group with small tumor size (diameter > 5 cm),[26] no haplogroups were found associated with tumor size (Table 5). Notably, we found that mean AFP levels and tumor size were lowest in patients with haplogroup N9a and were top ranked in patients with haplogroup D5 and B5, respectively (Figures S1A and S1B). Taken together, our results suggest that mtDNA haplogroup N9a is not associated with clinical characteristics of HCC.

Table 4. Association between mtDNA Haplogroup and Serum AFP Level

mtDNA Haplogroup	AFP[a] High (n = 117) [n (%)]	AFP[a] Low (n = 256) [n (%)]	Multivariate OR (95% CI)	Multivariate p Value
A	11 (9.4)	19 (7.4)	1.005 (0.417–2.418)	0.992
B4	16 (13.7)	18 (7.0)	1.651 (0.728–3.743)	0.230
B5	3 (2.6)	11 (4.3)	0.493 (0.124–1.965)	0.316
CZ	5 (4.3)	20 (7.8)	0.418 (0.140–1.248)	0.118
D4	31 (26.5)	59 (23.0)	1.0	
D5	10 (8.5)	24 (9.4)	0.743 (0.309–1.790)	0.508
F1	12 (10.3)	15 (5.9)	1.536 (0.630–3.743)	0.345
G	5 (4.3)	15 (5.9)	0.729 (0.238–2.230)	0.579
M7	4 (3.4)	20 (7.8)	0.356 (0.110–1.157)	0.086
N9a	1 (0.9)	6 (2.3)	0.354 (0.040–3.128)	0.350
Y	0 (0.0)	1 (0.4)	0.000 (0.000)	1.000
Others	19 (16.2)	48 (18.8)	0.787 (0.391–1.584)	0.502
Age			0.966 (0.945–0.987)	0.002*
HBV/HCV			1.179 (0.542–2.565)	0.679
Gender			0.826 (0.452–1.510)	0.534

[a]15 patients were excluded and AFP level and HBV/HCV infection status were not available for 14 patients and 1 patient, respectively.
*p < 0.004 (0.05/12), adjusted p value with Bonferroni correction while 12 haplogroups were studied; CI, confidence interval; OR, odds ratio.

N9a Cybrids and Non-N9a (B5, D4, and D5) Cybrids Feature Distinct Transcriptome Profiles and Neoplastic Activity

To investigate the contribution of N9a in tumorigenesis, we generated five cybrid cells by fusion of mtDNA-lacking Rho zero human osteosarcoma 143B cells with platelet containing mtDNA haplogroup N9a (N9a10a and N9a1) and non-N9a cybrids (B5, D4, and D5), respectively. *In vitro* neoplastic assay revealed that growth rate of N9a cybrids was significantly slower than non-N9a cybrids (Figure 3A). Moreover, the number of colonies formed in N9a cybrids was significantly less than non-N9a cybrids (Figure 3B). These data suggest that cells with haplogroup N9a have lower tumorigenic activity than non-N9a cybrids.

Next, we sought to identify the molecular mechanisms responsible for the negative correlation between haplogroup N9a and HCC. We analyzed the transcriptome of two N9a cybrids (N9a10a and N9a1) and three non-N9a cybrids (B5, D4, and D5). Multiple genes were differentially expressed between N9a cybrids and non-N9a cybrids; the transcription of 2,748 genes increased and the transcription of 3,185 genes decreased ($p < 0.05$, $|\log_2 FC| > 1$) (Figure 4A). Using the Kyoto Encyclopedia of Genes and Genomes (KEGG) database as a reference, gene set enrichment analysis (GSEA) revealed that 49

Table 5. Association between mtDNA Haplogroup and Tumor Size

	Tumor Size[a]		Multivariate	
mtDNA Haplogroup	Large (n = 142) [n (%)]	Small (n = 170) [n (%)]	OR (95% CI)	p Value
A	15 (10.6)	12 (7.1)	1.470 (0.602–3.587)	0.397
B4	9 (6.3)	21 (12.4)	0.475 (0.191–1.184)	0.110
B5	9 (6.3)	3 (1.8)	3.599 (0.884–14.653)	0.074
CZ	7 (4.9)	12 (7.1)	0.626 (0.221–1.773)	0.378
D4	37 (26.1)	42 (24.7)	1.0	
D5	11 (7.7)	17 (10.0)	0.761 (0.311–1.860)	0.549
F1	9 (6.3)	12 (7.1)	0.806 (0.302–2.150)	0.667
G	6 (4.2)	7 (4.1)	1.059 (0.323–3.473)	0.925
M7	7 (4.9)	13 (7.6)	0.614 (0.218–1.724)	0.354
N9a	3 (2.1)	4 (2.4)	0.819 (0.167–4.007)	0.805
Y	0 (0.0)	1 (0.6)	0.000 (0.000)	1.000
Others	29 (20.4)	26 (15.3)	1.302 (0.647–2.622)	0.460
Age			0.981 (0.960–1.003)	0.089
HBV/HCV			0.687 (0.330–1.428)	0.314
Gender			0.720 (0.402–1.289)	0.269

CI, confidence interval; OR, odds ratio.
[a]76 patients were excluded and tumor size and HBV/HCV infection status were not available for 72 patients and 4 patients, respectively.

pathways were differentially regulated in N9a cybrids compared to non-N9a cybrids (Data S1). Nearly 50% of the KEGG pathways identified by GSEA were associated with cellular metabolism (Figure 4B), and most of these, including fatty acid metabolism and fatty acid synthesis, were downregulated. Further, we found that increased activity in the oxidative phosphorylation (OXPHOS) pathway was positively correlated with the prevalence of haplogroup N9a in study participants (Figure 4B). However, in a previous study, we demonstrated that increased expression of nuclear DNA-encoded but not mtDNA-encoded OXPHOS subunits in N9a cybrids may compensate for diminished mitochondrial function (Figures 4C and 4D), and suggested that the N9a haplogroup exhibited lower mitochondrial OXPHOS activity than non-N9a cybrids.[11] We performed GSEA using the Reactome and CGP (chemical and genetic perturbations) gene sets from the Molecular Signatures Database (MSigDB) collection. Moreover, positive regulatory machinery of cell-cycle progressions was less activated in N9a cybrids than non-N9a cybrids. Alternatively, cell-cycle checkpoint pathways (including both p53-dependent and p53-independent checkpoints) were more activated in N9a cybrids than non-N9a cybrids (Figure 4E). Overall, the analysis found that the genetic profiles of non-N9a cybrids positively correlated with liver cancer invasion, progression, and recurrence, whereas the genetic profiles of N9a cybrids negatively correlated with liver cancer (Figure 4F).

DISCUSSION

The association of mtDNA haplogroups with HCC in China varied by geographical region. In northern China, haplogroup M and its defining SNP, m.T489C, were positively associated (albeit with marginal significance) with later age-at-onset of HCC,[18] although haplogroup M7 was negatively associated with HCC in eastern China.[19] In this study, we found that haplogroup N9a was negatively associated with HCC in northern China, but neither haplogroup M7 nor macro-mtDNA haplogroup M (defined by m.10400C > T) was associated with HCC in the same region.

Previous studies found that haplogroup N9a was negatively associated with type 2 diabetes (T2D) and other metabolic syndromes in Japan and Korea;[27] however, this association was not found in Taiwan.[28] Recently, we identified N9a as a risk factor for T2D in the southern Chinese population,[11] which suggests that haplogroup N9a may play a geographic specific role in the development of metabolic disease. While HCC is positively co-related with metabolic diseases including non-alcoholic fatty liver disease (NAFLD) and T2D,[29,30] it is reasonable that the haplogroup N9a may play the same role in the incidence of HCC. Because other factors may differentially affect the role of haplogroup N9a on T2D in different regions,[11,27,28] it is difficult to speculate whether haplogroup N9a plays a consensus or opposite role in HCC and T2D in northern China. Further studies on the association between haplogroup N9a and metabolic diseases in northern China may help. Notably, we found that mean AFP and mean tumor volume are top ranked in patients with haplogroup D5 and haplogroup B5, indicating that haplogroup B5 and D5 may play a role in the disease progression of HCC. Interestingly, our previous report showed that cybrid with haplogroup D5 is positively associated with cancer progression, such as cancer cell reproductive viability, transforming potential, and migration.[10,31] Although associations between haplogroup B5 and cancers were not previously reported, a positive association between haplogroup B5 and Alzheimer's disease susceptibility was reported.[14] These suggested that haplogroup B5 and D5 may play a role in the progression of HCC. However, due to the limit of sample size and lack of follow-up of patients with HCC, further studies with large sample size and clinical characteristics with time trends are needed to fully address the association between mtDNA haplogroups and clinical features.

Both increased and decreased mitochondrial OXPHOS functions were shown to promote tumorigenesis in different cancers with distinct mechanisms.[32,33] For some mtDNA haplogroups (e.g., haplogroup D5 in breast cancer), lower mitochondrial function may promote tumorigenesis due to altered mitochondrial signals including reactive oxygen species and NAD^+/NADH, which are responsible for overactivation of mitochondrial retrograde signaling.[10] Although haplogroup N9a was positively associated with OXPHOS function in GSEA using microarray data in one study, this conclusion was not supported by the following mitochondrial function study.[34] In our recent study, we confirmed that N9a is positively associated with OXPHOS-related genes expression;[11] however, we found that mitochondrial function in N9a cybrids was lower than in non-N9a cybrids,[11] suggesting that the positive correlation between OXPHOS GSEA and haplogroup N9a may be a means of compensating for reduced mitochondrial function in N9a cybrids. In this study, a positive association between OXPHOS GSEA and haplogroup N9a was confirmed

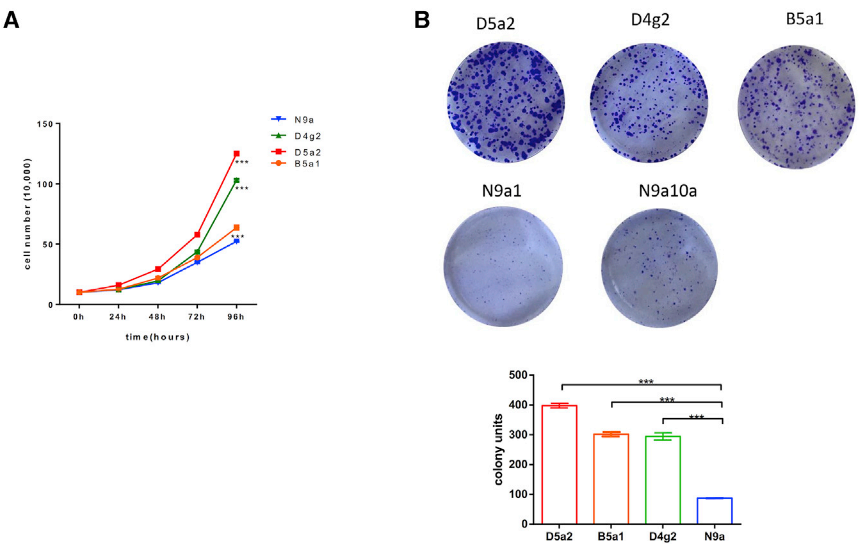

Figure 3. Haplogroup N9a Have Lower Tumorigenic Activity than Non-N9a (B5a1, D5a2, D4g2) Cybrids
(A) Cell proliferation curve of N9a and non-N9a cybrids (n = 3). (B) Colony formation assay of N9a and non-N9a cybrids (n = 3). Data are presented by means ± SEM, and ***p < 0.001.

further by using two N9a cybrids and three non-N9a cybrids (B5, D4, and D5) (Figure 4B). Therefore, it is likely that haplogroup N9a plays a protective role in HCC development by decreasing mitochondrial function. Increased mitochondrial function was shown to promote tumorigenesis by generating ATP for cancer-related kinase activation,[33] supplying tricarboxylic-acid-cycle-derived amino acid for endothelial cell proliferation during angiogenesis,[35] and driving epithelial-mesenchymal transition progress for metastasis.[32] In this study, we did see an increased metastatic activity in non-N9a cybrids compared with N9a cybrids by using MSigDB-based GSEA (Data S2). Furthermore, GSEA using KEGG revealed that half (24/49) of enriched pathways are metabolic pathways (Data S1). Given that change of metabolic profiles is one of the key features in tumorigenesis, we believe that N9a and non-N9a haplogroups play different role in HCC incidence. However, we do not know how cellular metabolism pathways are regulated by different performance of mitochondrial function in N9a and non-N9a haplogroup containing cells. Further studies on the causal relationship between OXPHOS function and cellular metabolism pathways are necessary to completely reveal the protective role of haplogroup N9a in HCC.

In conclusion, we identified a negative correlation between mtDNA haplogroup N9a and HCC prevalence and tumor volume in patients with HCC. We also demonstrated that cells containing haplogroup N9a and non-haplogroup N9a exhibit distinct metabolic profiles, which may contribute to the negative correlation between N9a and the probability of developing HCC. Additional studies are needed to fully characterize the metabolic basis for the effects of haplogroup N9a on HCC incidence and progression.

MATERIALS AND METHODS

Study Participants

From March 2018 to July 2019, we recruited 388 patients with HCC (mean ± SD 55.68 ± 0.55, median age 55, range 12–88) from the Henan Provincial People's Hospital (n = 209, mean ± SD 56.18 ± 0.79, median age 57, range 12–88), the First Affiliated Hospital of Zhengzhou University (n = 115, mean ± SD 55.23 ± 0.94, median = 55, range 27–77), and the Henan Provincial Cancer Hospital (n = 64, mean ± SD 54.86 ± 1.32, median = 53.5, range 34–73) to participate in the study. Each diagnosis of HCC was confirmed histologically, either immediately after surgery or with a subsequent liver biopsy. Meanwhile, we recruited 511 geographically matched, asymptomatic control participants (mean ± SD 56.19 ± 0.59, median = 58, range 19–87) with no history of cancer or of other diseases associated with mitochondrial defects (e.g., diabetes and hypertension), from The Physical Examination Center of Henan Provincial People's Hospital from May 2019 to July 2019. In the patient group, 338 patients are HBV or HCV positive (320 patients are HBV positive, 16 patients are HCV positive, and 2 patients are HBV+HCV positive), 43 patients are HBV/HCV negative, and HBV/HCV infection history was missing in 7 patients. All control subjects are HBV/HCV negative. A detailed characteristic of age and sex of the patients and control subjects was described in Table S10. Informed consent was obtained from the subjects enrolled in this study, and the study was approved by the Ethics Committee of Wenzhou Medical University and all three hospitals we mentioned above.

mtDNA Sequencing and Genotyping

Genomic DNA was extracted from peripheral blood using a standard SDS lysis protocol. Two primer pairs were designed to amplify mtDNA fragments, which contain major diagnostic SNPs of the Asian mtDNA haplogroup: L15975F: 5′-CTCCACCATTAGCACC CAAAGC-3′ and H794R: 5′-AGGCTAAGCGTTTTGAGCTG-3′; L9967F: 5′-TCTCCATCTATTGATGAGGGTCT-3′ and H10858R: 5′-AATTAGGCTGTGGGTGGTTG-3′. An additional sequencing primer, 299F 5′-GGTGGAAATTTTTTGTTATG-3′, was designed to detect a potential poly C gap between mt16184 and mt16193. PCR was performed using a PCR Amplification Kit (TaKaRa, Tokyo, Japan) with a S1000 Thermal Cycler (Bio-Rad, Hercules, CA, USA) under the following conditions: pre-denaturation at 95°C for 5 minutes, followed by 35 cycles of (94°C for 30 s, 57°C for 30 s, and 72°C for 40 s), with a final extension at 72°C for 4 min. Sanger sequencing was performed using ABI 3730XL (Thermo Fisher Scientific, Waltham, MA, USA). SNPs of each participant were identified by comparing sequences with the revised Cambridge Reference Sequence (rCRS) using CodonCode Aligner 3.0.1 (CodonCode Corporation, Centerville, VA, USA). A mtDNA haplogroup was

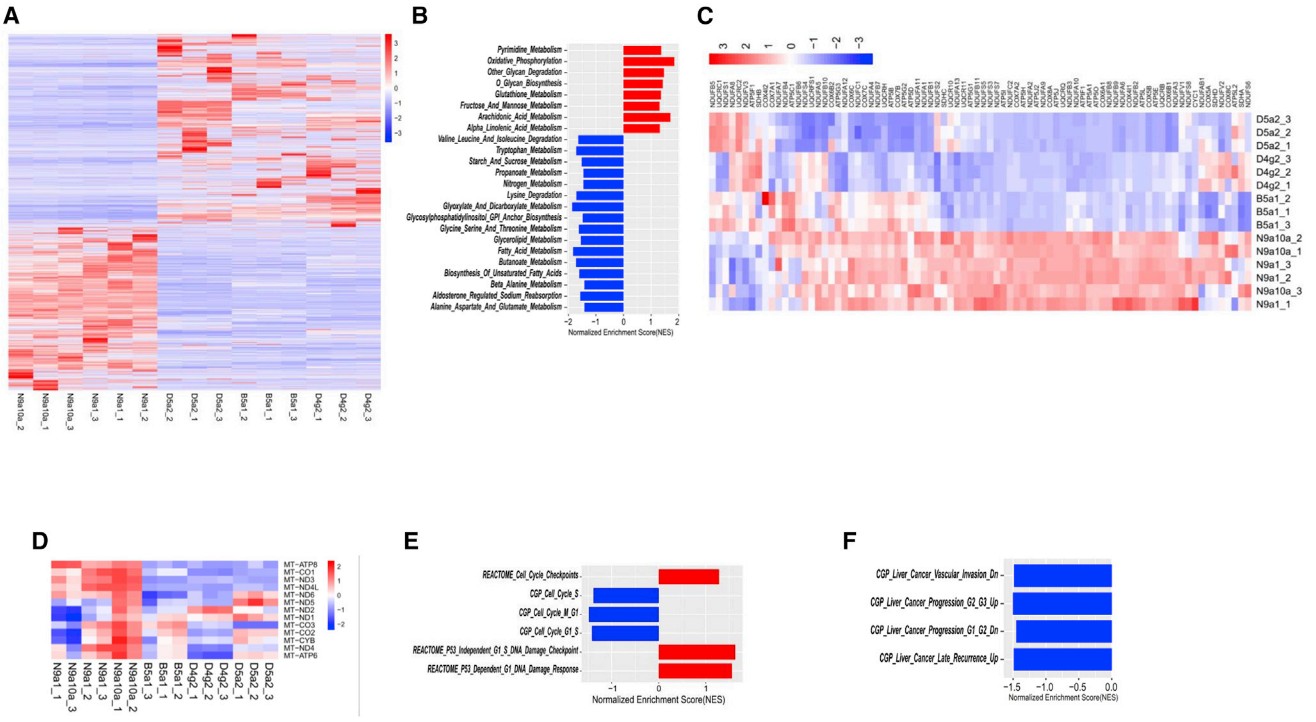

Figure 4. N9a and Non-N9a (B5a1, D5a2, D4g2) Cybrids Feature Distinct Transcriptome Profiles

(A) Heatmap of RNA-seq data showing the differentially expressed genes (DEGs) between N9a and non-N9a cybrids ($p < 0.05$, $|log_2FC| > 1$). (B) GSEA enrichment using KEGG database showing metabolic pathways are differently activated between N9a and non-N9a cybrids. (C and D) Heatmap showing transcriptional changes of nuclear-encoded (C) and mitochondrially encoded (D) OXPHOS subunits in N9a and non-N9a cybrids. (E) GSEA enrichment of cell-cycle related events in N9a and non-N9a cybrids using KEGG, Reactome, and GCP from MsigDB. (F) GSEA enrichment of liver cancer related events in N9a and non-N9a cybrids using GCP.

identified for each participant using MitoTool,[36] and visually confirmed by comparing SNPs from the D-loop of ND3 and ND4L, with the diagnostic SNPs from the east Asian mtDNA haplogroup tree.[37–39] If haplogroup identification was still inconclusive, additional information was obtained by restriction fragment-length polymorphism (RFLP) analysis at sites: 3,010 (BccI), 4,833 (HhaI), 5,178 (AluI), and 9,824 (HinfI) and by testing for the 9 bp deletion at the COII-tRNA^{lys} junction. mtDNA sequence variations and RFLP results for each individual can be found in Data S3.

In Vitro Tumorigenesis Assay

For cell proliferation assay, a total of 100,000 cells were cultured in 6-well plate, and the number of cells was counted for 24 h, 48 h, 72 h, and 96 h. For colony formation assay, 1,000 cells were cultured in 6-well plate for 2 weeks and then fixed with 4% paraformaldehyde (30525-89-4,Yonghua, Shanghai, China) for 30 min, followed by staining with 0.1% crystal violet solution (C0121, Beyotime, Shanghai, China) for 30 min, and then washed three times with PBS buffer. ImageJ v2.4.1.7 was used to analyze the number of clones.

RNA Sequencing and Analysis of Gene-Expression Data

Two N9a cybrids (N9a10a and N9a1) and three non-N9a (B5, D4, and D5) cybrids were generated in our previous study.[40] To evaluate the differences between the gene-expression profiles of N9a cybrids and non-N9a cybrids, we isolated total RNA from each group of cybrids (in triplicate) using a RNeasy Mini Extraction kit (QIAGEN, Valencia, CA, USA), and mRNA from 20 μg of the total RNA was purified using poly-T-attached magnetic beads. After fragmenting the mRNA, first-strand cDNA was synthesized and sequenced using an Illumina HiSeq 2000 platform (Illumina, San Diego, CA, USA) as described previously.[11] Clean reads were obtained by deleting adaptor-only sequences and low-quality sequences. Sequence comparison was carried out using STAR (v2.5.1b) against 1000 Genomes Build 37 Decoy 5.[30] The number of annotated clean reads of each gene was analyzed and normalized as reads per kilobase per million reads (RPKM).[31] Genes were considered differentially expressed if their transcript level changed at least 2-fold ($p < 0.05$) compared with controls. GSEA was performed using the KEGG and Reactome databases as references. To evaluate the enrichment of liver cancer-related pathways, we performed GSEA using gene sets of CGP from the MSigDB as references.[41] The minimum and maximum genes number were 30–200 for GSEA. Pathways were considered significantly enriched with false discovery rate < 0.25 and $p < 0.05$.

Statistical Analysis

In this case-control study, statistical analysis of mtDNA haplogroup and HCC was performed in three levels: macro-haplogroups M and

N, major branches of haplogroups M and N according to the phylogenetic tree of Han Chinese,[38] and haplogroup with frequency of >5% in either the control group or the patient group; these were included to evaluate the potential association between common mtDNA haplogroups and HCC. All other haplogroups with frequencies of <5% were collectively evaluated as "other haplogroups." PCA of the mtDNA haplogroup frequencies was conducted by the R package to show the clustering pattern of patients with HCC and control subjects, as well as reported Han Chinese population across China (Table S11). The minimal sample size study was determined by using Quanto software in the study of associations between mtDNA SNPs and patients with HCC.[42] FPRP analysis was calculated by the R package.[43] Multivariate logistical regression analysis was applied to adjust for disparities in age and gender between the patient group and control group. We found that for each mtDNA haplogroup, HCC was a dependent variable, whereas age, sex, and genotype were independent variables. Because there was high numerical variability among the 14 mtDNA haplogroups included in the analysis (i.e., A, G, M7, M9, M10, B4, B5, CZ, D4, D5, F1, N9a, Y, and "other haplogroups"), we applied dummy coding. A Bonferroni correction indicating $p < 0.004$ (0.05/14) was considered statistically significant when evaluating these haplogroups. To examine the relationships among liver function biomarkers and the mtDNA haplogroups, we applied multivariate logistical regression analysis to adjust for risks associated with age, sex, and HBV infection status. One-way ANOVA was used to analyze the differences in mean AFP expression and volume among ten mtDNA haplogroups. *In vitro* tumorigenesis assays were analyzed using two-tailed Student's t test. All statistical analyses were performed using SPSS 21.0 (IBM, Armonk, NY, USA) unless otherwise indicated.

SUPPLEMENTAL INFORMATION

Supplemental Information can be found online at https://doi.org/10.1016/j.omtn.2019.09.001.

AUTHOR CONTRIBUTIONS

L.S., M.J., and H.F. conceived and designed the study. S.H., M.L., J.W., Y.Z., and J.L. collected the samples, performed the PCR, and aligned sequences. Q.Z. and J.L. performed the RNA-sequencing experiment and the following bioinformatical analysis. L.S., M.J., and H.F. supervised the work and wrote the manuscript.

ACKNOWLEDGMENTS

This work was supported by the National Natural Science Foundation of China (31671486 and 31501156), Key Discipline of Zhejiang Province in Medical Technology (First Class, Category A), and Zhejiang Provincial Natural Science Foundation of China (LY16H060001).

REFERENCES

1. Chen, J.G., and Zhang, S.W. (2011). Liver cancer epidemic in China: past, present and future. Semin. Cancer Biol. *21*, 59–69.

2. Tummala, K.S., Brandt, M., Teijeiro, A., Graña, O., Schwabe, R.F., Perna, C., and Djouder, N. (2017). Hepatocellular Carcinomas Originate Predominantly from Hepatocytes and Benign Lesions from Hepatic Progenitor Cells. Cell Rep. *19*, 584–600.

3. Song, B.J., Akbar, M., Abdelmegeed, M.A., Byun, K., Lee, B., Yoon, S.K., and Hardwick, J.P. (2014). Mitochondrial dysfunction and tissue injury by alcohol, high fat, nonalcoholic substances and pathological conditions through post-translational protein modifications. Redox Biol. *3*, 109–123.

4. Kim, S.J., Khan, M., Quan, J., Till, A., Subramani, S., and Siddiqui, A. (2013). Hepatitis B virus disrupts mitochondrial dynamics: induces fission and mitophagy to attenuate apoptosis. PLoS Pathog. *9*, e1003722.

5. Reznik, E., Wang, Q., La, K., Schultz, N., and Sander, C. (2017). Mitochondrial respiratory gene expression is suppressed in many cancers. eLife *6*, 6.

6. Ni, Z., He, J., Wu, Y., Hu, C., Dai, X., Yan, X., Li, B., Li, X., Xiong, H., Li, Y., et al. (2018). AKT-mediated phosphorylation of ATG4B impairs mitochondrial activity and enhances the Warburg effect in hepatocellular carcinoma cells. Autophagy *14*, 685–701.

7. Szczepanowska, K., and Trifunovic, A. (2017). Origins of mtDNA mutations in ageing. Essays Biochem. *61*, 325–337.

8. Vyas, S., Zaganjor, E., and Haigis, M.C. (2016). Mitochondria and Cancer. Cell *166*, 555–566.

9. Bannwarth, S., Procaccio, V., Lebre, A.S., Jardel, C., Chaussenot, A., Hoarau, C., Maoulida, H., Charrier, N., Gai, X., Xie, H.M., et al. (2013). Prevalence of rare mitochondrial DNA mutations in mitochondrial disorders. J. Med. Genet. *50*, 704–714.

10. Ma, L., Fu, Q., Xu, B., Zhou, H., Gao, J., Shao, X., Xiong, J., Gu, Q., Wen, S., Li, F., et al. (2018). Breast cancer-associated mitochondrial DNA haplogroup promotes neoplastic growth via ROS-mediated AKT activation. Int. J. Cancer *142*, 1786–1796.

11. Fang, H., Hu, N., Zhao, Q., Wang, B., Zhou, H., Fu, Q., Shen, L., Chen, X., Shen, F., and Lyu, J. (2018). mtDNA Haplogroup N9a Increases the Risk of Type 2 Diabetes by Altering Mitochondrial Function and Intracellular Mitochondrial Signals. Diabetes *67*, 1441–1453.

12. Zhang, A.M., Jia, X., Bi, R., Salas, A., Li, S., Xiao, X., Wang, P., Guo, X., Kong, Q.P., Zhang, Q., and Yao, Y.G. (2011). Mitochondrial DNA haplogroup background affects LHON, but not suspected LHON, in Chinese patients. PLoS ONE *6*, e27750.

13. Ji, Y., Zhang, A.M., Jia, X., Zhang, Y.P., Xiao, X., Li, S., Guo, X., Bandelt, H.J., Zhang, Q., and Yao, Y.G. (2008). Mitochondrial DNA haplogroups M7b1′2 and M8a affect clinical expression of leber hereditary optic neuropathy in Chinese families with the m.11778G–>a mutation. Am. J. Hum. Genet. *83*, 760–768.

14. Bi, R., Zhang, W., Yu, D., Li, X., Wang, H.Z., Hu, Q.X., Zhang, C., Lu, W., Ni, J., Fang, Y., et al. (2015). Mitochondrial DNA haplogroup B5 confers genetic susceptibility to Alzheimer's disease in Han Chinese. Neurobiol. Aging *36*, 1604, e7–16.

15. Kazuno, A.A., Munakata, K., Nagai, T., Shimozono, S., Tanaka, M., Yoneda, M., Kato, N., Miyawaki, A., and Kato, T. (2006). Identification of mitochondrial DNA polymorphisms that alter mitochondrial matrix pH and intracellular calcium dynamics. PLoS Genet. *2*, e128.

16. Suissa, S., Wang, Z., Poole, J., Wittkopp, S., Feder, J., Shutt, T.E., Wallace, D.C., Shadel, G.S., and Mishmar, D. (2009). Ancient mtDNA genetic variants modulate mtDNA transcription and replication. PLoS Genet. *5*, e1000474.

17. Latorre-Pellicer, A., Moreno-Loshuertos, R., Lechuga-Vieco, A.V., Sánchez-Cabo, F., Torroja, C., Acín-Pérez, R., Calvo, E., Aix, E., González-Guerra, A., Logan, A., et al. (2016). Mitochondrial and nuclear DNA matching shapes metabolism and healthy ageing. Nature *535*, 561–565.

18. Guo, Z., Yang, H., Wang, C., and Liu, S. (2012). Mitochondrial DNA haplogroup M is associated with late onset of hepatocellular carcinoma. Exp. Ther. Med. *3*, 499–502.

19. Chen, C., Ba, Y., Li, D., Du, X., Lia, X., Yang, H., An, J., Xing, J., Yang, H., Dong, G., and Guo, X. (2017). Genetic variations of mitochondrial genome modify risk and prognosis of hepatocellular carcinoma patients. Clin. Res. Hepatol. Gastroenterol. *41*, 378–385.

20. Li, S., Wan, P., Peng, T., Xiao, K., Su, M., Shang, L., Xu, B., Su, Z., Ye, X., Peng, N., et al. (2016). Associations between sequence variations in the mitochondrial DNA D-loop region and outcome of hepatocellular carcinoma. Oncol. Lett. *11*, 3723–3728.

21. Salas, A., García-Magariños, M., Logan, I., and Bandelt, H.J. (2014). The saga of the many studies wrongly associating mitochondrial DNA with breast cancer. BMC Cancer *14*, 659.

22. Salas, A., and Elson, J.L. (2015). Mitochondrial DNA as a risk factor for false positives in case-control association studies. J. Genet. Genomics *42*, 169–172.

23. Hu, S.P., Du, J.P., Li, D.R., and Yao, Y.G. (2014). Mitochondrial DNA haplogroup confers genetic susceptibility to nasopharyngeal carcinoma in Chaoshanese from Guangdong, China. PLoS ONE *9*, e87795.

24. Nojiri, S., Fujiwara, K., Shinkai, N., Endo, M., and Joh, T. (2014). Evaluation of hepatocellular carcinoma development in patients with chronic hepatitis C by EOB-MRI. World J. Hepatol. *6*, 930–938.

25. Ryder, S.D.; British Society of Gastroenterology (2003). Guidelines for the diagnosis and treatment of hepatocellular carcinoma (HCC) in adults. Gut *52* (*Suppl 3*), iii1–iii8.

26. Molden, J., Kao, J., and Shah, A.P. (2008). Treatment of high-burden thrombus in a large right coronary artery. J. Invasive Cardiol. *20*, E48–E51.

27. Fuku, N., Park, K.S., Yamada, Y., Nishigaki, Y., Cho, Y.M., Matsuo, H., Segawa, T., Watanabe, S., Kato, K., Yokoi, K., et al. (2007). Mitochondrial haplogroup N9a confers resistance against type 2 diabetes in Asians. Am. J. Hum. Genet. *80*, 407–415.

28. Liou, C.W., Chen, J.B., Tiao, M.M., Weng, S.W., Huang, T.L., Chuang, J.H., Chen, S.D., Chuang, Y.C., Lee, W.C., Lin, T.K., and Wang, P.W. (2012). Mitochondrial DNA coding and control region variants as genetic risk factors for type 2 diabetes. Diabetes *61*, 2642–2651.

29. Ray, K. (2018). NAFLD-HCC: target cholesterol. Nat. Rev. Gastroenterol. Hepatol. *15*, 390.

30. Lagiou, P., Kuper, H., Stuver, S.O., Tzonou, A., Trichopoulos, D., and Adami, H.O. (2000). Role of diabetes mellitus in the etiology of hepatocellular carcinoma. J. Natl. Cancer Inst. *92*, 1096–1099.

31. Fang, H., Shen, L., Chen, T., He, J., Ding, Z., Wei, J., Qu, J., Chen, G., Lu, J., and Bai, Y. (2010). Cancer type-specific modulation of mitochondrial haplogroups in breast, colorectal and thyroid cancer. BMC Cancer *10*, 421.

32. LeBleu, V.S., O'Connell, J.T., Gonzalez Herrera, K.N., Wikman, H., Pantel, K., Haigis, M.C., de Carvalho, F.M., Damascena, A., Domingos Chinen, L.T., Rocha, R.M., et al. (2014). PGC-1alpha mediates mitochondrial biogenesis and oxidative phosphorylation in cancer cells to promote metastasis. Nat. Cell Biol. *16*, 992–1003, 1–15.

33. Zhou, C., Sun, H., Zheng, C., Gao, J., Fu, Q., Hu, N., Shao, X., Zhou, Y., Xiong, J., Nie, K., et al. (2018). Oncogenic HSP60 regulates mitochondrial oxidative phosphorylation to support Erk1/2 activation during pancreatic cancer cell growth. Cell Death Dis. *9*, 161.

34. Hwang, S., Kwak, S.H., Bhak, J., Kang, H.S., Lee, Y.R., Koo, B.K., Park, K.S., Lee, H.K., and Cho, Y.M. (2011). Gene expression pattern in transmitochondrial cytoplasmic hybrid cells harboring type 2 diabetes-associated mitochondrial DNA haplogroups. PLoS ONE *6*, e22116.

35. Diebold, L.P., Gil, H.J., Gao, P., Martinez, C.A., Weinberg, S.E., and Chandel, N.S. (2019). Mitochondrial complex III is necessary for endothelial cell proliferation during angiogenesis. Nat Metab *1*, 158–171.

36. Fan, L., and Yao, Y.G. (2011). MitoTool: a web server for the analysis and retrieval of human mitochondrial DNA sequence variations. Mitochondrion *11*, 351–356.

37. Kong, Q.P., Bandelt, H.J., Sun, C., Yao, Y.G., Salas, A., Achilli, A., Wang, C.Y., Zhong, L., Zhu, C.L., Wu, S.F., et al. (2006). Updating the East Asian mtDNA phylogeny: a prerequisite for the identification of pathogenic mutations. Hum. Mol. Genet. *15*, 2076–2086.

38. Yao, Y.G., Kong, Q.P., Bandelt, H.J., Kivisild, T., and Zhang, Y.P. (2002). Phylogeographic differentiation of mitochondrial DNA in Han Chinese. Am. J. Hum. Genet. *70*, 635–651.

39. Kivisild, T., Tolk, H.V., Parik, J., Wang, Y., Papiha, S.S., Bandelt, H.J., and Villems, R. (2002). The emerging limbs and twigs of the East Asian mtDNA tree. Mol. Biol. Evol. *19*, 1737–1751.

40. Zhou, H., Nie, K., Qiu, R., Xiong, J., Shao, X., Wang, B., Shen, L., Lyu, J., and Fang, H. (2017). Generation and Bioenergetic Profiles of Cybrids with East Asian mtDNA Haplogroups. Oxid. Med. Cell. Longev. *2017*, 1062314.

41. Liberzon, A., Subramanian, A., Pinchback, R., Thorvaldsdóttir, H., Tamayo, P., and Mesirov, J.P. (2011). Molecular signatures database (MSigDB) 3.0. Bioinformatics *27*, 1739–1740.

42. Gauderman, W.J. (2002). Sample size requirements for matched case-control studies of gene-environment interaction. Stat. Med. *21*, 35–50.

43. Wacholder, S., Chanock, S., Garcia-Closas, M., El Ghormli, L., and Rothman, N. (2004). Assessing the probability that a positive report is false: an approach for molecular epidemiology studies. J. Natl. Cancer Inst. *96*, 434–442.

OMTN, Volume *18*

Supplemental Information

Mitochondrial DNA Haplogroup N9a Negatively Correlates with Incidence of Hepatocellular Carcinoma in Northern China

Shixuan Hua, Meinan Li, Qiongya Zhao, Junyi Wang, Yaping Zhou, Jiangtao Liu, Hezhi Fang, Minghua Jiang, and Lijun Shen

Table S1. Analysis of association between mitochondrial haplogroups and HBV/HCV positive HCC

MtDNA haplogroup	Patients (n = 338)	Controls (n = 511)	OR (95% CI)	*P* value
Macro-haplogroup				
A	29 (8.6)	42 (8.2)	0.890 (0.510-1.555)	0.683
B	52 (15.4)	72 (14.1)	0.986 (0.625-1.555)	0.951
D	111 (32.8)	140 (27.4)	1.0	
F	33 (9.8)	63 (12.3)	0.647 (0.389-1.075)	0.093
G	16 (4.7)	22 (4.3)	0.872 (0.425-1.788)	0.708
M7	22 (6.5)	35 (6.8)	0.756 (0.410-1.393)	0.370
M8	30 (8.9)	46 (9.0)	0.691 (0.402-1.187)	0.181
M9	3 (0.9)	6 (1.2)	0.600 (0.140-2.575)	0.492
M10	6 (1.8)	12 (2.3)	0.555 (0.196-1.572)	0.268
N9	6 (1.8)	35 (6.8)	0.191 (0.076-0.479)	0.000*
Others	30 (8.9)	38 (7.4)	0.962 (0.548-1.691)	0.894
Age			0.998 (0.987-1.010)	0.783
Gender			0.280 (0.202-0.388)	0.000*
Sub-haplogroup				
B4	32 (9.5)	36 (7.0)	1.167 (0.650-2.094)	0.605
B5	15 (4.4)	28 (5.5)	0.899 (0.434-1.863)	0.775
CZ	22 (6.5)	37 (7.2)	0.657 (0.352-1.228)	0.188
D4	77 (22.8)	101 (19.8)	1.0	
D5	32 (9.5)	38 (7.4)	1.151 (0.644-2.057)	0.636
F1	24 (7.1)	37 (7.2)	0.771 (0.417-1.425)	0.407
N9a	5 (1.5)	26 (5.1)	0.213 (0.077-0.590)	0.003*
Y	1 (0.3)	9 (1.8)	0.158 (0.019-1.317)	0.088
Others[a]	79 (23.4)	122 (23.9)	0.841 (0.548-1.291)	0.429
Age			0.999 (0.987-1.011)	0.869
Gender			0.281 (0.202-0.390)	0.000*

* indicated $P < 0.004$ (0.05/14), adjusted *P* value with Bonferroni correction while 14 haplogroups were studied; CI, confidence interval; OR, odds ratio; values in ( ) are the percentage (%) of samples; [a] Haplogroups with frequencies less than 5% in both patients and controls.

Table S2. Analysis of association between mitochondrial haplogroups and HBV/HCV negative HCC

MtDNA haplogroup	Patients (n = 43)	Controls (n = 511)	OR (95% CI)	*P* value
Macro-haplogroup				
A	2 (4.7)	42 (8.2)	0.385 (0.085-1.744)	0.215
B	2 (4.7)	72 (14.1)	0.240 (0.054-1.074)	0.062
D	17 (39.5)	140 (27.4)	1.0	
F	6 (14.0)	63 (12.3)	0.768 (0.287-2.059)	0.600
G	5 (11.6)	22 (4.3)	1.894 (0.626-5.730)	0.258
M7	2 (4.7)	35 (6.8)	0.460 (0.101-2.101)	0.316
M8	5 (11.6)	46 (9.0)	0.786 (0.272-2.274)	0.657
M9	0 (0.0)	6 (1.2)	0.000 (0.000)	0.999
M10	0 (0.0)	12 (2.3)	0.000 (0.000)	0.999
N9	2 (4.7)	35 (6.8)	0.403 (0.088-1.845)	0.242
Others	2 (4.7)	38 (7.4)	0.429 (0.094-1.948)	0.273
Age			1.018 (0.993-1.043)	0.160
Gender			0.470 (0.233-0.949)	0.035
Sub-haplogroup				
B4	2 (4.7)	36 (7.0)	0.404 (0.087-1.883)	0.248
B5	0 (0)	28 (5.5)	0.000 (0.000)	0.998
CZ	3 (7.0)	37 (7.2)	0.491 (0.132-1.834)	0.290
D4	14 (32.6)	101 (19.8)	1.0	
D5	3 (7.0)	38 (7.4)	0.497 (0.133-1.857)	0.298
F1	5 (11.6)	37 (7.2)	0.879 (0.292-2.644)	0.819
N9a	2 (4.7)	26 (5.1)	0.454 (0.096-2.156)	0.321
Y	0 (0.0)	9 (1.8)	0.000 (0.000)	0.999
Others[a]	5 (11.6)	100 (19.6)	0.334 (0.115-0.970)	0.044
Age			1.028 (0.995-1.046)	0.110
Gender			0.440 (0.237-0.970)	0.041

CI, confidence interval; OR, odds ratio; values in ( ) are the percentage (%) of samples; [a] Haplogroups with frequencies less than 5% in both patients and controls.

Table S3. False-positive report probability analysis for significant finding

SNP	OR (95%CI)	*P*	Statistical power[a]	Prior probability				
				**0.25**	**0.1**	0.01	0.001	0.0001
C16257A	0.290 (0.123-0.685)	0.005	0.802	**0.018**	**0.056**	0.308	0.856	0.978

CI, confidence interval; OR, odds ratio; [a] statistical power was computed by the OR and *p* value; the odds ratio of 0.2 for protective effect.

Table S4. Analysis of association between mitochondrial DNA SNPs and HBV/HCV positive HCC

mtSNP	Patients (n = 338)	Controls (n = 511)	OR (95% CI)	*P* value
m.150C>T	64 (18.9)	94 (18.4)	1.076 (0.780-1.484)	0.656
m.249delA	42 (12.4)	85 (16.6)	0.647 (0.446-0.941)	0.023
m.10397A>G	34 (10.1)	38 (7.4)	1.559 (1.007-2.416)	0.047
m.10398A>G	224 (66.3)	316 (61.8)	1.220 (0.936-1.590)	0.140
m.10400C>T	202 (59.8)	278 (54.4)	1.147 (0.888-1.480)	0.294
m.16129G>A	54 (16.0)	106 (20.7)	0.649 (0.462-0.912)	0.013
m.16189T>C	124 (36.7)	147 (28.8)	1.486 (1.137-1.943)	0.004*
m.16223C>T	211 (62.4)	293 (57.3)	1.174 (0.906-1.521)	0.224
m.16257C>A	5 (1.5)	26 (5.1)	0.262 (0.107-0.643)	0.003*
m.16362T>C	165 (48.8)	214 (41.9)	1.317 (1.021-1.698)	0.034

*indicated $P < 0.005$ (0.05/10), adjusted P value with Bonferroni correction while 10 SNPs were studied; CI, confidence interval; OR, odds ratio; values in ( ) are the percentage (%) of samples.

Table S5. Analysis of association between mitochondrial DNA SNPs and HBV/HCV negative HCC

mtSNP	Patients (n = 43)	Controls (n = 511)	OR (95% CI)	*P* value
m.150C>T	3 (0.07)	94 (18.4)	0.262 (0.080-0.857)	0.027
m.249delA	6 (14.0)	85 (16.6)	0.858 (0.392-1.875)	0.700
m.10397A>G	3 (7.0)	38 (7.4)	0.951 (0.326-2.776)	0.927
m.10398A>G	26 (60.5)	316 (61.8)	1.049 (0.589-1.868)	0.870
m.10400C>T	26 (60.5)	278 (54.4)	1.449 (0.815-2.577)	0.206
m.16129G>A	7 (16.3)	106 (20.7)	0.754 (0.360-1.582)	0.456
m.16189T>C	10 (23.3)	147 (28.8)	0.728 (0.372-1.424)	0.354
m.16223C>T	27 (62.8)	293 (57.3)	1.413 (0.787-2.539)	0.247
m.16257C>A	2 (4.7)	26 (5.1)	0.674 (0.155-2.928)	0.598
m.16362T>C	22 (51.2)	214 (41.9)	1.665 (0.951-2.915)	0.074

CI, confidence interval; OR, odds ratio; values in ( ) are the percentage (%) of samples.

Table S6. Association between mtDNA haplogroups and serum AST level

MtDNA haplogroup	AST rise		Multivariate	
	Yes (n = 185) [n (%)]	No (n = 195) [n (%)]	OR (95% CI)	*P* value
A	15 (8.1)	16 (8.2)	1.097 (0.480-2.508)	0.827
B4	17 (9.2)	17 (8.7)	1.202 (0.541-2.671)	0.651
B5	7 (3.8)	8 (4.1)	1.031 (0.339-3.134)	0.957
CZ	15 (8.1)	10 (5.1)	1.779 (0.715-4.428)	0.215
D4	40 (21.6)	51 (26.2)	1.0	
D5	19 (10.3)	16 (8.2)	1.413 (0.637-3.132)	0.395
F1	16 (8.6)	13 (6.7)	1.602 (0.684-3.750)	0.278
G	7 (3.8)	14 (7.2)	0.701 (0.256-1.916)	0.488
M7	12 (6.5)	12 (6.2)	1.179 (0.472-2.943)	0.724
N9a	3 (1.6)	4 (2.1)	1.058 (0.222-5.039)	0.944
Y	0 (0.0)	1 (0.5)	0.000 (0.000)	1.000
Others	34 (18.4)	33 (16.9)	1.309 (0.689-2.487)	0.411
Age			0.978 (0.959-0.997)	0.023
HBV/HCV			1.354 (0.690-2.655)	0.378
Gender			1.072 (0.633-1.815)	0.795

CI, confidence interval; OR, odds ratio; eight patients are not available for AST clinical data.

Table S7. Association between mtDNA haplogroups and serum ALT level.

MtDNA haplogroup	ALT rise		Multivariate	
	Yes (n = 120) [n (%)]	No (n = 259) [n (%)]	OR (95% CI)	*P* value
A	11 (9.2)	19 (7.3)	1.228 (0.511-2.950)	0.646
B4	12 (10.0)	22 (8.5)	1.157 (0.500-2.675)	0.734
B5	5 (4.2)	10 (3.9)	1.087 (0.334-3.533)	0.890
CZ	9 (7.5)	16 (6.2)	1.106 (0.434-2.817)	0.833
D4	29 (24.2)	62 (23.9)	1.0	
D5	9 (7.5)	26 (10.0)	0.751 (0.309-1.826)	0.528
F1	8 (6.7)	21 (8.1)	0.761 (0.300-1.931)	0.565
G	5 (4.2)	16 (6.2)	0.692 (0.229-2.089)	0.513
M7	7 (5.8)	17 (6.6)	0.892 (0.330-2.408)	0.821
N9a	2 (1.7)	5 (1.9)	0.854 (0.154-4.734)	0.857
Y	0 (0.0)	1 (0.4)	0.000 (0.000)	1.000
Others	23 (19.2)	44 (17.0)	1.147 (0.582-2.257)	0.692
Age			0.990 (0.970-1.010)	0.322
HBV/HCV			1.022 (0.490-2.127)	0.955
Gender			0.577 (0.315-1.057)	0.075

CI, confidence interval; OR, odds ratio; nine patients are not available for ALT clinical data.

Table S8. Association between mtDNA haplogroups and serum ALB level.

MtDNA haplogroup	ALB down		Multivariate	
	Yes (n = 178) [n (%)]	No (n = 202) [n (%)]	OR (95% CI)	*P* value
A	13 (7.3)	18 (8.9)	0.975 (0.424-2.241)	0.953
B4	18 (10.1)	16 (7.9)	1.500 (0.676-3.330)	0.319
B5	7 (3.9)	8 (4.0)	1.180 (0.390-3.571)	0.769
CZ	13 (7.3)	12 (5.9)	1.439 (0.588-3.524)	0.425
D4	40 (22.5)	51 (25.2)	1.0	
D5	20 (11.2)	15 (7.4)	1.809 (0.816-4.013)	0.145
F1	9 (5.1)	20 (9.9)	0.567 (0.231-1.389)	0.214
G	7 (3.9)	14 (6.9)	0.588 (0.215-1.607)	0.301
M7	15 (8.4)	9 (4.5)	2.226 (0.876-5.657)	0.093
N9a	7 (3.9)	0 (0.0)	1912182463 (0.000)	0.999
Y	1 (0.6)	0 (0.0)	2526260229 (0.000)	1.000
Others	28 (15.7)	39 (19.3)	0.914 (0.480-1.741)	0.785
Age			1.018 (0.998-1.038)	0.076
HBV/HCV			0.822 (0.415-1.628)	0.575
Gender			0.902 (0.530-1.536)	0.704

CI, confidence interval; OR, odds ratio; eight patients are not available for ALB clinical data.

Table S9. Association between mtDNA haplogroups and serum TBIL level.

MtDNA haplogroup	TBIL rise		Multivariate	
	Yes (n = 86) [n (%)]	No (n = 294) [n (%)]	OR (95% CI)	*P* value
A	7 (8.1)	24 (8.2)	0.902 (0.336-2.421)	0.838
B4	9 (10.5)	25 (8.5)	1.133 (0.454-2.825)	0.789
B5	4 (4.7)	11 (3.7)	1.109 (0.313-3.925)	0.873
CZ	5 (5.8)	20 (6.8)	0.738 (0.243-2.236)	0.591
D4	21 (24.4)	70 (23.8)	1.0	
D5	3 (3.5)	32 (10.9)	0.294 (0.081-1.070)	0.063
F1	7 (8.1)	22 (7.5)	1.018 (0.377-2.751)	0.972
G	4 (4.7)	17 (5.8)	0.886 (0.263-2.985)	0.845
M7	5 (5.8)	19 (6.5)	0.839 (0.275-2.555)	0.757
N9a	3 (3.5)	4 (1.4)	2.984 (0.594-14.978)	0.184
Y	1 (1.2)	0 (0.0)	7065260239 (0.000)	1.000
Others	17 (19.8)	50 (17.0)	1.120 (0.531-2.363)	0.766
Age			0.985 (0.962-1.008)	0.200
HBV/HCV			2.400 (0.882-6.530)	0.087
Gender			0.590 (0.289-1.203)	0.147

CI, confidence interval; OR, odds ratio; eight patients are not available for TBIL clinical data.

Table S10. Characteristics of the participants

Characteristic	Patients (n = 388)	Controls (n = 511)
Age (years)		
10-20	1 (0.3)	1 (0.2)
21-30	3 (0.8)	21 (4.1)
31-40	24 (6.2)	55 (10.8)
41-50	103 (26.5)	77 (15.1)
51-60	123 (31.7)	136 (26.6)
61-70	103 (26.5)	152 (29.7)
71-80	27 (7.0)	58 (11.4)
81-90	4 (1.0)	11 (2.2)
Gender		
Male	308 (79.4)	282 (55.2)
Female	80 (20.6)	229 (44.8)

values in ( ) are the percentage (%) of samples

Table S11. Statistics of Han populations from different regions

Geography	Population ID	Locality	No. of samples	References
Northern China	HCC	Zhengzhou, Henan	388	This study
	CON	Zhengzhou, Henan	511	This study
	HA	Henan	198	[1]
	YB	Yanbian, Jilin	51	[2]
	GS	Gansu	44	[2]
	DL	Dalian, Liaoning	50	[2]
	FC	Fengcheng, Liaoning	50	[2]
	CF	Chifeng, Neimeng	44	[2]
	XN	Xining, Qinghai	41	[2]
	QD	Qingdao, Shandong	50	[2]
	TA	Taian, Shandong	76	[2]
	XA	Xi'an, Shanxi	53	[2]
	UR	Urumqi, Xinjiang	47	[2]
	QH	Qinghai	78	[3]
	ZB	Zibo, Shandong	50	[3]
Southern China	HF	Hefei, Anhui	42	[2]
	CT	Changtin, Fujian	54	[2]
	GZ	Guangzhou, Guangdong	68	[2]
	ZJ	Zhanjiang, Guangdong	30	[2]
	TL	Tianlin, Guangxi	26	[2]
	WH	Wuhan, Hubei	42	[2]
	CS	Changsha, Hunan	16	[2]
	NJ	Nanjing, Jiangsu	67	[2]
	NC	Nanchang, Jiangxi	23	[2]
	WC	Weicheng, Sichuan	70	[2]
	KM	Kunming, Yunnan	42	[2]
	YNHZ	Huize, Yunnan	59	[2]
	HK	HongKong	20	[3]
	TW1	Taiwan	66	[3]
	TW2	Taiwan	155	[3]
	SH	Shanghai	120	[3]
	ZJWZ	Wenzhou, Zhejiang	974	[4]

1. Su B, Xiao C, Deka R, Seielstad MT, Kangwanpong D, Xiao J, Lu D, Underhill P, Cavalli-Sforza L, Chakraborty R *et al*: *Y chromosome haplotypes reveal prehistorical migrations to the Himalayas. Human genetics* 2000, **107**(6):582-590.
2. Wen B, Li H, Lu D, Song X, Zhang F, He Y, Li F, Gao Y, Mao X, Zhang L *et al*: *Genetic evidence supports demic diffusion of Han culture. Nature* 2004, **431**(7006):302-305.
3. Yao YG, Kong QP, Bandelt HJ, Kivisild T, Zhang YP: *Phylogeographic differentiation of mitochondrial DNA in Han Chinese. Am J Hum Genet* 2002, **70**(3):635-651.
4. Fang H, Hu N, Zhao Q, Wang B, Zhou H, Fu Q, Shen L, Chen X, Shen F, Lyu J: *mtDNA Haplogroup N9a Increases the Risk of Type 2 Diabetes by Altering Mitochondrial Function and Intracellular Mitochondrial Signals. Diabetes* 2018, **67**(7):1441-1453.
